# Molecular Interaction-Based Exploration of the Broad Spectrum Efficacy of a *Bacillus thuringiensis* Insecticidal Chimeric Protein, Cry1AcF

**DOI:** 10.3390/toxins11030143

**Published:** 2019-03-02

**Authors:** Maniraj Rathinam, Karthik Kesiraju, Shweta Singh, Vinutha Thimmegowda, Vandna Rai, Debasis Pattanayak, Rohini Sreevathsa

**Affiliations:** 1 ICAR-National Research Centre on Plant Biotechnology, New Delhi 110012, India; rmani607@gmail.com (M.R.); kartikkesiraju@gmail.com (K.K.); shwetaasinngh@gmail.com (S.S.); vandnarai2006@gmail.com (V.R.); debasispattanayak@yahoo.co.in (D.P.); 2 Division of Biochemistry, ICAR-Indian Agricultural Research Institute, New Delhi 110012, India; vinuthabiochem@gmail.com

**Keywords:** chimeric protein, insecticidal proteins, insect resistance, modeling, protein-protein docking

## Abstract

*Bacillus thuringiensis* insecticidal proteins (*Bt* ICPs) are reliable and valuable options for pest management in crops. Protein engineering of *Bt* ICPs is a competitive alternative for resistance management in insects. The primary focus of the study was to reiterate the translational utility of a protein-engineered chimeric Cry toxin, Cry1AcF, for its broad spectrum insecticidal efficacy using molecular modeling and docking studies. In-depth bioinformatic analysis was undertaken for structure prediction of the Cry toxin as the ligand and aminopeptidase1 receptors (APN1) from *Helicoverpa armigera* (*Ha*APN1) and *Spodoptera litura* (*Sl*APN1) as receptors, followed by interaction studies using protein-protein docking tools. The study revealed feasible interactions between the toxin and the two receptors through H-bonding and hydrophobic interactions. Further, molecular dynamics simulations substantiated the stability of the interactions, proving the broad spectrum efficacy of Cry1AcF in controlling *H. armigera* and *S. litura.* These findings justify the utility of protein-engineered toxins in pest management.

## 1. Introduction

Battling insects for a better tomorrow is the need of the hour. Transgenic technology has emerged as a promising option to help farmers mitigate insect pressure on crops, the most competent option being the use of *Bacillus thuringiensis* (*Bt*) insecticidal proteins (ICPs). The global adoption of genetically engineered (GE) crop technology continues at unassuming rates since its introduction. There has been a considerable increase in the acreage of transgenic crops since 1996, making it the fastest adopted crop technology in the recent past [1].

A plethora of *cry* genes are unceasingly being identified by scientists worldwide for plausible introgression into crop plants through transgenesis. An array of genes as well as crops have been exploited to demonstrate the sustained use of the technology [2,3,4,5,6,7]. However, one of the major concerns of plant scientists is the development of resistance by insects towards Cry proteins encoding genes [5,8]. To circumvent this, various strategies are being followed with protein-engineered chimeric toxins being one of the viable approaches [9,10,11,12,13]. The hypothesis is that production of protein-engineered chimeric toxins by domain swapping and other strategies would not only result in the toxin emerging with a broad spectrum efficacy but also to facilitate in the avoidance of resistance in the insects towards Cry toxins. One such chimeric toxin, Cry1AcF (patent no 237912), synthesized by swapping domains between *cry1Ac* and *cry1F*, demonstrated synergism and improved efficacy towards *H. armigera* [14]. This chimeric gene has been successfully validated for efficacy in a number of economically important crops like castor, chick pea, groundnut, field bean, and pigeon pea, etc., against two insect pests, *H. armigera* and *S. litura* [15,16,17,18,19]. Through transgenesis, we could demonstrate that Cry1AcF exhibited higher insecticidal activity against the two insect herbivores as evidenced by reduced larval growth, increased insect mortality, and reduced damage to the leaves. Validation of the broad spectrum efficacy of Cry1AcF by demonstrating its molecular interaction with the concomitant receptors will be worthwhile.

Bioinformatic tools are being effectively used in the area of biotechnology for in depth understanding of various molecular interactions. These tools have made structural biology much advanced and the work sophisticated, precise, and simple with high accuracy and worldwide acceptability. Elucidation of protein structures, protein-protein docking, and other tools to study interactions between the receptors in the insects and the Cry proteins have been widely demonstrated [7,9,20,21,22,23].

It is a known fact that the cry1 series of proteins recognize the aminopeptidase receptors 1 (APN1) in the insect gut [24,25]. Individual Cry toxins are usually toxic to only a few species within an order, and receptors on midgut epithelial cells have been shown to be critical determinants of Cry specificity. The best characterized receptors for lepidopterans are those belonging to the classes of aminopeptidase N (APN) receptors and cadherin-like receptors. The major emphasis of this study was to justify the utility of *cry1AcF* gene against two pests, and more emphatically to demonstrate the ability of the chimeric toxin to bind and interact with APN1s from both *H. armigera* and *S. litura* by exploiting in silico modeling and molecular interactions. This understanding would aid in the development of a stable platform to explain the broad spectrum efficacy of chimeric toxins developed through protein engineering and their use in crop improvement programs.

## 2. Results and Discussion

**Figure 1:**
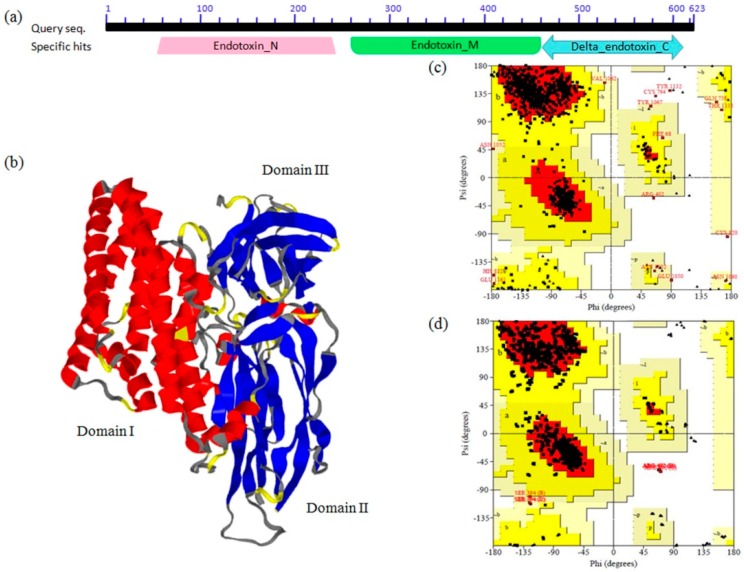
Structure prediction and validation of Cry1AcF. (**a**) Putative domains in Cry1AcF as predicted by NCBI Conserved Domain search; (**b**) 3-D structure of Cry1AcF; (**c**) Ramachandran plot for the Cry1AcF model as determined by PROCHECK; (**d**) Ramachandran plot for the template 4ARY 1A model as determined by PROCHECK.

With the increasing popularity and use of transgenics for the management of insects, addressing resistance development in the insect pests is imperative. The use of Cry proteins, especially those having broad spectrum efficacy, are important tools for insect resistance management [26]. Several strategies have been proposed to combine *B. thuringiensis* toxins [27], and a number of these fusion/chimeric/protein-engineered toxins with broad spectrum efficacy against insect pests have been introgressed into crop plants. Domains I and II of the gene *cry1AcF*, belonged to *cry1Ac* whereas domain III was from *cry1F*. A NCBI-CDD (Conserved Domain Database) search demonstrated Cry1AcF to be a 623 amino acid (aa) protein consisting of three functional domains: N-terminal domain I (48–251 aa), domain II (259–461 aa), and C-terminal domain III (463–608 aa) (Figure 1a). Similarly, INTERPROSCAN analysis of APN1 receptors from *H. armigera* and *S. litura* depicted that the receptors belonged to the aminopeptidase-type family and consisted of peptidase M1 domain and ERAP1-like C-terminal domain.

### 2.1. Molecular Modeling and Validation

Homology or comparative modeling forms a remarkable option for the development of theoretical 3-D protein models, especially when a clear homology is seen between the sequences of target protein and an elucidated structure. This goes with the assumption that the tertiary structure of two proteins could be similar if there is sequence similarity. The protein model for Cry1AcF was predicted using the modeler server against the chosen template, 4ARY chain A (a *cry1Ac Bacillus thuringiensis* delta endotoxin belonging to Kurstaki sub species), which had 85% identity with the target protein (Figure 1b). The quality of the Cry1AcF model as well as the template was validated through the structural evaluation program PROCHECK (Table 1), and Ramachandran plots provided the analysis of peptide dihedral angles of misfolded proteins into allowed and non-allowed regions. Compared with the template 4ARY chain A, the built 3-D model had a similar Ramachandran plot, which showed that the predicted model was reliable in terms of its backbone conformation (Figure 1c,d) and supported by the placement of 98% of residues in the allowed regions. The quality of the developed Cry1AcF model was further validated by the ERRAT score of 77.193, indicating the non-randomly distributed atoms, which are considered to be reliable. The Verify-3D results of Cry1AcF model showed that 92.56% of the amino acids had an average 3D-1D score of >0.2, indicating the dependability of the proposed model. The energy profile of the proposed model and the Z-score value (a measure of model quality as it measures the total energy of the structures) were obtained using the program ProSA, which calculates the interaction energy per residue using a distance-based pair potential. ProSA analysis of the Cry1AcF model demonstrated a Z-score of −9.69 (where the negative Z-score energy reflects reliability of the model), reflecting the quality of the model. These results together validated the quality of the Cry1AcF model (Table 2), albeit with variation between the proteins in Domain III, (cry 1AcF and 4ARY chain A shared only 44% of similarity in domain III), that did not hamper the structure prediction.

**Figure 2:**
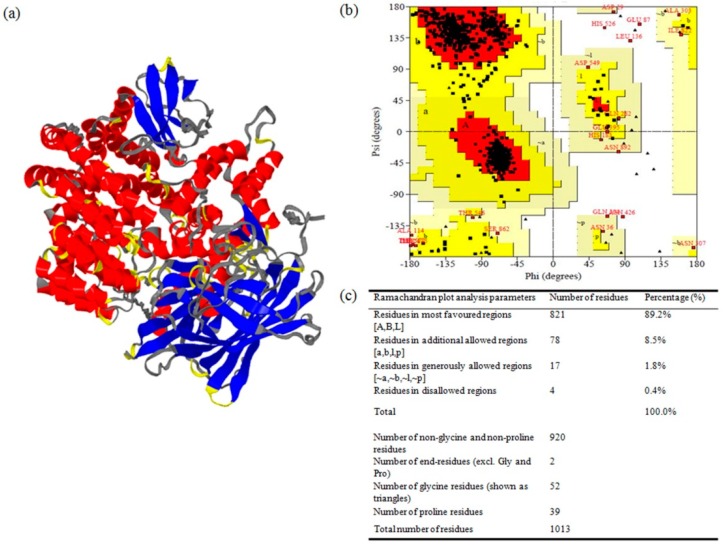
Structure prediction and validation of aminopeptidase1 receptors (APN1) from *H. armigera* (*Ha*APN1). (**a**) 3-D structure of *Ha*APN1; (**b**) Ramachandran plot for the APN1 model as determined by PROCHECK; (**c**) Ramachandran plot statistics to validate the modeled *Ha*APN1.

**Figure 3:**
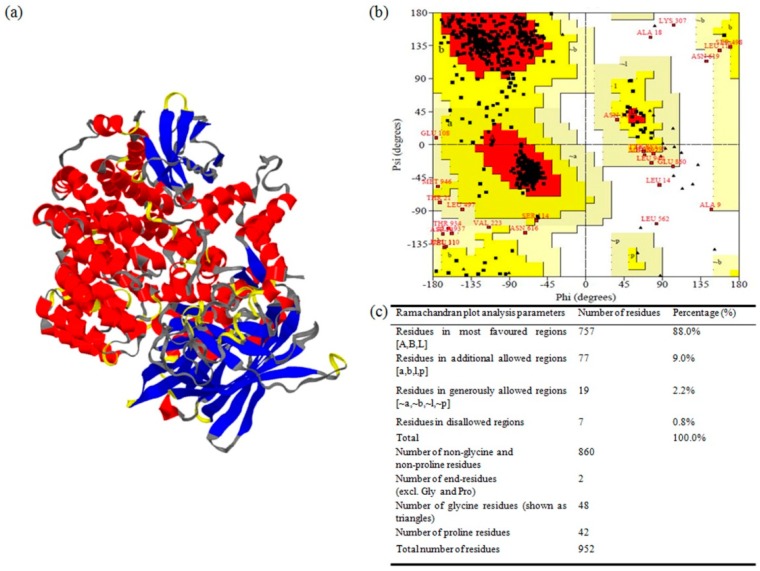
Structure prediction and validation of APN1 from *S. litura* (*Sl*APN1). (**a**) 3-D structure of *Sl*APN1; (**b**) Ramachandran plot for the APN1 model as determined by PROCHECK; (**c**) Ramachandran plot statistics to validate the modeled *Sl*APN1.

Contrary to homology modeling, prediction of 3-D structures for proteins that lack homologous protein structures deposited in the Protein Data Bank (PDB) is generally carried out by protein threading, which is also known as fold recognition. In the present study, considering the low homology of *Ha*APN1 and *Sl*APN1 proteins with that of the other experimental/available structures from PDB database, modeling was done using the threading approach at Modeller ITASSER server (Figure 2a and Figure 3a). The generated models were further compared with secondary structures in order to reiterate the quality of modeling (Supplementary File S1). The typical four-domain well-separated structure of APN receptors was evident in our study and could be identified by their unique structures [28]. The pictorial representation of the secondary structures of APN proteins, with respect to their sequences showed that the N-terminal end or domain I of both the proteins were composed of β-sheets while the C-terminal region or domain IV was composed of α-helices. Domains II and III consisted of α-helices and β-sheets, respectively. The comparative analysis of secondary structures of both the receptor proteins indicated the presence of 33 helix moieties in *Ha*APN1 compared to 40 in *Sl*APN1. Analysis also demonstrated the presence of disulphide bridges at two regions, one in the H23 helix region and the other between two helices, H24 and H27, in *Ha*APN1. Similarly, two disulfide bonds were observed in *Sl*APN1 at the H29 and H30–H31 regions. The major difference between the APN1 proteins in the two insect herbivores was observed to be in the threonine-rich region in the C-terminal end (Supplementary File S1). Ramachandran plot analysis to assess stoichiometric quality of modeled APN1 proteins demonstrated the location of >97% of amino acids in the allowed regions, showing the stability of the two modeled proteins (Figure 2b,c and Figure 3b,c). The quality of the developed models for APN1 receptors from *H. armigera* and *S. litura* were further supported by an ERRAT score of 65.8 and 67, respectively, indicating that the non-randomly distributed atoms are considered to be reliable. Similarly, Verify-3D results showed that 79.86% and 83.40% of the respective amino acids had an average 3D-1D score of >0.2, indicating the reliability of the developed models (Table 2). ProSA analysis demonstrated a Z-score of −10.21 and −10.89, respectively, which reflected the quality of the models (Table 2).

### 2.2. Docking and Interaction Analysis

**Figure 4:**
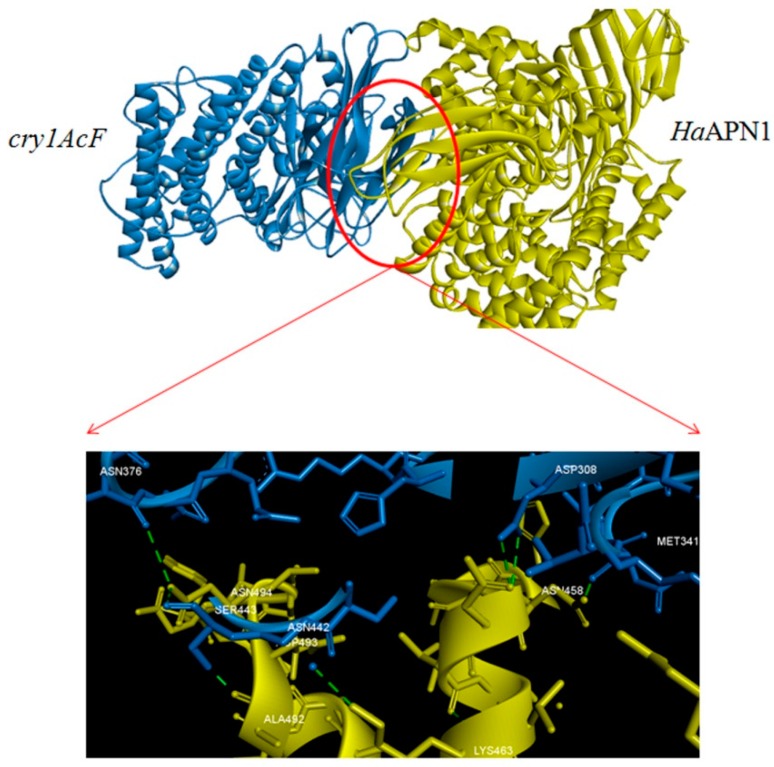
Molecular interaction between Cry1AcF and *Ha*APN1 (insect: specific region of interaction between the two proteins).

**Figure 5:**
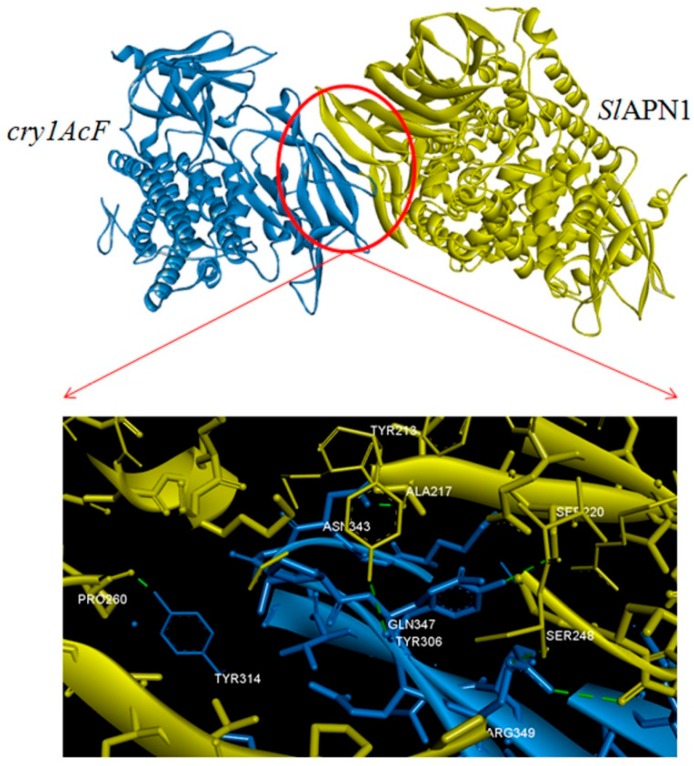
Molecular interaction between Cry1AcF and *Sl*APN1 (insect: specific region of interaction between the two proteins).

Protein-protein interaction analysis or protein-protein docking is one of the important aspects of structural biology that aids in the prediction of the structure of a protein-protein complex based on the structures of the individual proteins. To understand this, computational strategies have evolved with time, taking into consideration the growing knowledge on protein structure and interaction [29]. The docking of Cry1AcF and APN1 proteins from *H. armigera* and *S. litura,* respectively, was performed using PatchDock, a protein-protein docking software. The APN1 receptor proteins were considered as B chain, and Cry1AcF protein in both the cases as A chain. Cry proteins comprises three domains: a seven-helix-bundle domain (DI) involved in membrane insertion and pore formation; a three-antiparallel-β-sheet domain (DII) considered as the most probable candidate for receptor binding; and a β-sandwich domain (DIII) considered as a multifunctional domain involved in structural integrity, membrane penetration, ion channel function, and a major determinant of receptor binding [5,9]. Our study portrayed that the chosen chimeric Cry protein Cry1AcF interact with the receptors in the region of domain II in case of both the insect specific APN1 receptors (Figure 4 and Figure 5). The interacting molecules as viewed by Discovery studio [30] showed that conspicuous interactions in both the complexes were seen between amino acids of domain II in Cry1AcF and APN1 receptors. Amino acid Gln347 of Cry1AcF-domain II was seen to play a prominent role (Figure 4 and Figure 5; Table 3 and Table 4) in both the interactions, indicating that Cry1AcF could be interacting with the receptor from both insects in similar competence and leading to effectiveness against the two insect pests.

**Figure 6:**
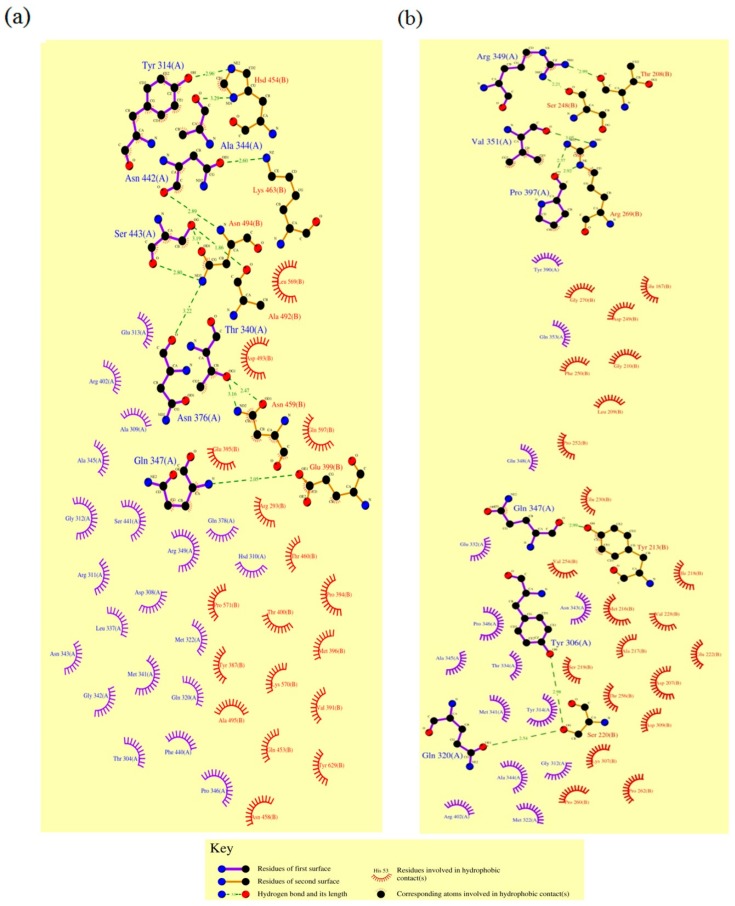
Demonstration of interaction between the chimeric Cry1AcF (A chain) and APN1 receptors (B chain) by Dimplot. (**a**) and (**b**) Dimplot images of interaction between Cry1AcF and APN1 from *H. armigera* and *S. litura*, respectively.

Further, this interaction at the molecular level is apparent in the present study as seen by Dim plot (domain-domain interaction plot) analysis with respect to hydrogen bond interactions (shown as green dotted lines in Figure 6) between the amino acid residues of APN1 receptors and Cry protein residues (Table 3 and Table 4). Hydrogen-bonding groups on interacting surfaces are majorly responsible for molecular recognition and hydrogen bond interactions conferring rigidity to the protein structure. It was observed in both the complexes that the distance between H-bond interactions ranged from 1.8 Å to 3.05 Å. As per the Watson and Crick categorization, donor-acceptor distances of 2.2–2.5 Å are considered as “strong or covalent bonds” whereas, 2.5–3.2 Å are “moderate and mostly electrostatic”, and 3.2–4.0 Å are “weakly electrostatic” [31]. In the present study, 11 and 8 H bonds (Table 3 and Table 4) were detected in the *Ha*APN1-Cry1AcF and *Sl*APN1-Cry1AcF interactions, respectively. In both the complexes, hydrogen bonding is seen between the amino acids in domain II of Cry1AcF and the receptors (Table 3 and Table 4), confirming the involvement of domain II in receptor recognition. Distinct combinations of amino acids involved in H bond formations were observed in both the interactions. The study demonstrated that Asn, Ser, and Thr were involved in H bond formation in Cry1AcF whereas Asn was the key player (Figure 6; Table 3) in *Ha*APN1. However, in the case of the *Sl*APN1-Cry1AcF complex, Arg and Ser were prominent. Gln347 from Cry1AcF was found to interact with both the receptors, reiterating the broad spectrum recognition and action of Cry1AcF. Similarly, hydrophobic interactions are also important for the folding and stability of proteins as it allows the protein to decrease in surface area and reduce undesirable interactions with water. It was evident from Dimplot that hydrophobic interactions were maximum in the *Ha*APN1-Cry1AcF complex (Figure 6a). Amino acid residues like Tyr314, Gln320, Met322, Thr340, Ala344, Pro346, Gln347, and Arg349 from domain II of Cry1AcF were found to be involved in hydrophobic interactions with *Ha*APN1 whereas Gly312, Tyr314, Asn343, Gln347, and Gln348 were found to be interacting with *Sl*APN1 (Figure 6b). Furthermore, in the case of the *Ha*APN1-Cry1AcF complex, amino acid residues in the region 380–495 in domain II were seen to be involved, whereas residues from 210–306 (a region spanning domain I and II) were found to be interacting with Cry1AcF in the *Sl*APN1 complex. This clearly indicated distinct interaction patterns between Cry1AcF protein and its two receptors, demonstrating efficacy of the protein-engineered toxin against both the pests through definite molecular interactions. The interactions seen in the present finding corroborated with the studies which revealed that APNs interact with Cry toxins through domain II (which acts as a catalytic region) [24,25,26]. Similarly, discrete regions of the receptors were found to be interacting with the Cry toxin (Figure 4 and Figure 5). In the *Ha*APN1-Cry1AcF complex, amino acids in the region, 399–494 were seen to be interacting whereas in the *Sl*APN1-Cry1AcF complex, amino acids from 208–269 were involved in the interaction. Asn 494 and Arg 269 were seen to play a prominent role in the complex formation of Cry1AcF with *Ha*APN1 and *Sl*APN1, respectively (Table 3 and Table 4). Corroborative evidences through mutation studies from other groups have also explicitly demonstrated the importance of domain II of Cry proteins in receptor recognition and toxicity [32,33].

### 2.3. Molecular Dynamic Simulations

**Figure 7:**
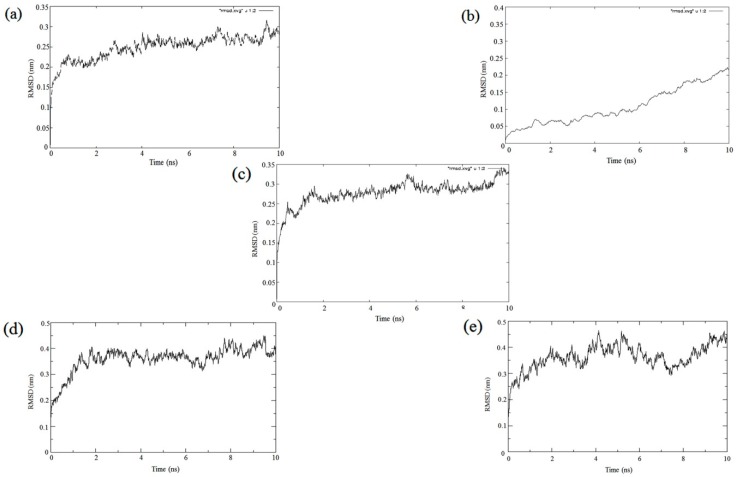
Root mean square deviation (RMSD) graphs of (**a**) Cry1AcF; (**b**) *Ha*APN1; (**c**) *Sl*APN1; (**d**) *Ha*APN1-Cry1AcF complex; and (**e**) *Sl*APN1-Cry1AcF complex.

Evidence for stability in proteins and protein complexes is an important aspect in structure biology. This can be understood by stability analyses under simulated conditions. Molecular dynamics simulation is carried out to determine the movement of Cα backbone atoms over a fixed time period. Root mean square deviation (RMSD) of Cα atoms in a molecule with respect to a reference structure was used to study the time points when conformation changes occur and stabilize. Analysis of the fluctuation of Cα coordinates in the modeled Cry1AcF and APN1 receptors as well as the complexes demonstrated stability under simulated conditions (Figure 7a–c). The stability of protein conformations can be relatively determined by deviations that have been produced during the course of simulation. Stable protein structures exhibit less deviation in comparison to the proteins that possess unstable structure. With respect to the individual proteins, the RMSD simulation graph (Figure 7a) for Cry1AcF showed that equilibrium was reached in 2 ns and maintained a 0.25 nm RMSD value up to 10 ns. In the case of *Ha*APN1 (Figure 7b), the equilibrium was obtained before 2ns with a RMSD value of 0.05–0.1 nm and was maintained up to 6 ns, and thereafter a shift in RMSD value of 0.15–0.2 nm was observed till 10 ns. The RMSD graph of *Sl*APN1 (Figure 7c) showed that the equilibrium was reached at about 0.2 ns with a RMSD value of 0.25–0.3 nm maintained up to 10 ns. Thus, it can be concluded that all the three predicted structures showed lower RMSD value within the range of 0.05–0.3 nm, confirming the quality of the predicted models to be appropriate for docking studies.

Further, the complex of Cry1AcF and *Ha*APN1 (Figure 7d) reached an equilibrium at 2 ns with a RMSD value of 0.3–0.4 nm and was maintained up to 10 ns. However, the Cry1AcF and *Sl*APN1 complex (Figure 7e) reached an equilibrium before 1 ns and maintained a RMSD value between 0.3–0.45 nm up to 10 ns. It was observed that RMSD variation in both the receptor complexes was not >0.2 nm when compared to their individual unbound forms, indicating that both the receptors formed a stable interaction with the Cry1AcF protein. Concretely, molecular dynamics demonstrated evidence for effective interaction and stability between Cry1AcF and both the receptors.

The study provided additional value to the already proven multi-pest efficacy of the protein-engineered toxin Cry1AcF through molecular interactions, corroborating the translational potential of a toxin using in silico studies. Molecular modeling and interaction studies ardently demonstrated that the chimeric Cry1AcF binds and interacts with the APN1 receptors of both *H. armigera* and *S. litura*, which otherwise is not exhibited by the parent toxins. The overall findings demonstrate the feasibility of protein engineering and domain swapping as efficient tools for management of multiple pests towards improved resistance management.

## 3. Materials and Methods

### 3.1. Source of Sequence and Primary Analysis

The chimeric *Bt* gene *cry1AcF* selected in the present study was developed and validated in-house (patent no. 237912). The APN1 receptors from *H. armigera* (Genebank ID-EU568875.1) and *S. litura* (Genebank ID-AF320764.1) were chosen for interaction analysis with the Cry protein. The InterProScan [34] tool was used to predict the protein family and the domain arrangement within the proteins. Conserved domains of the proteins were explored by using NCBI Conserved Domain Database (CDD) [35].

### 3.2. Molecular Modeling and Validation

The suitable template for homology modeling was investigated by the DELTABLAST (Domain Enhanced Lookup Time Accelerated BLAST) [36] search tool and used against Protein Data Bank (PDB) [37]. The 3-D structure of Cry1AcF was predicted using modeller server by considering the template with high similarity. The best model was chosen on the basis of the stereochemistry quality report generated using PROCHECK [38], a program that provides an assessment of the stereochemistry of the overall quality of the structure and identifies regions to be investigated. The ERRAT tool [39] was used for structure verification of non-randomly distributed atoms based on their energetic and geometric effect. The non-randomly distributed atoms in a protein structure are expected to be most reliable whereas more random atom distributions are observed in unreliable models. VERIFY-3D [40] was used to check the compatibility of the thus developed 3D-model of a protein to its own amino acid sequences. The ProSA [41] program tool was used in the refinement of theoretical models by comparing them with the available known native proteins. Modeling of the APN1 proteins of *H. armigera* and *S. litura* was performed using modeller package ITASSER [42] using the threading approach, considering the low homology of the receptors with that of available structures from the PDB database. In order to perform the modeling, the templates were searched in the sequence database at NCBI using PSI-BLAST [43]. Further, the secondary structures of the target sequences were predicted using PSIPRED [44]. The homologous sequences thus obtained were aligned with the target sequence and modeled. The stereochemically accepted model for both the insect receptors of APN1 was polished using the same approach used for the Cry protein. The selected models were further refined using molecular dynamics (MD) simulation.

### 3.3. Docking and Interaction Analysis

The docking of Cry1AcF-APN1 from both insects was performed using the PatchDock protein-protein docking software [45] and visualized with Discovery studio [30]. The PatchDock software, a geometry-based molecular docking algorithm, was used to find the docking conformations with Cry1AcF protein as the A chain and APN1 receptor proteins from both the insects as the B chain. The docking was performed using default parameters with the rigid body docking module, as the structures are unbound and treated as rigid bodies. They were rotated against each other without conformational changes, and their side chain conformation was adjusted to optimize the interference between the unbound monomers [46]. The best model for analysis of interaction was chosen on the basis of the geometric shape complementary score and the minimum energy. The hydrogen bond and non-bonded interactions between the two protein complexes were analyzed using Ligplot^+^ [47].

### 3.4. Molecular Dynamics (MD) Simulations of Cry1AcF and APN1 Receptors

MD simulations were performed to optimize the models developed with both the native proteins as well as the complexes. The simulation was performed with the GROMOS96 43A1 [48] force field in the GROMACS 5.0.7 [49] package on a high performance computer. The Simple Point Charge (SPC) model was used to represent water. Protonation state of ionizable groups in each of the proteins was chosen as appropriate for pH 7.0. During the simulation, all the atoms of the docked complex were surrounded by a cubical box of SPC water with pressure and temperature maintained constant throughout the simulation. The minimum distance between any atom of the protein and the box wall was 1.0 nm, and periodic boundary conditions were maintained in all the directions. Coulomb and van der Waals interactions within a shorter-range cutoff of 1 nm were evaluated at every time step. Longer-range Coulomb and van der Waals interactions were also maintained at 1 nm and were updated with every time step. A 50 ps molecular dynamics with position restraints on the protein (PRMD) was performed at 250 K using the leap-frog integrator. During the simulation, electrostatic interactions were calculated by the particle mesh Ewald (PME) algorithm and covalent bonds in the protein. The system stability and differences in trajectories, root mean square deviation (RMSD), and the energies of the system were analyzed using tools available with the GROMACS package.

## Figures and Tables

**Table 1:** Ramachandran plot statistics for Cry1AcF and the template 4ARY chain A used in homology modeling.

Ramachandran Plot Analysis Parameters	Template 4ARY_A1		Cry1AcF	
	Number of Residues	Percentage (%)	Number of Residues	Percentage (%)
Template 4ARY 1A				
Residues in most favored regions [A,B,L]	1843	91.3%	938	89.8%
Residues in additional allowed regions [a,b,l,p]	168	8.3%	91	8.7%
Residues in generously allowed regions [~a,~b,~l,~p]	3	0.1%	10	1%
Residues in disallowed regions	4	0.2%	5	0.5%
Total		100%		100%
Number of non-glycine and non-proline residues	2018		1044	
Number of end-residues (excl. Gly and Pro)	5		2	
Number of glycine residues (shown as triangles)	172		83	
Number of proline residues	120		53	
Total number of residues	2315		1182	

**Table 2:** Validation of the predicted protein models.

Homology Modeled Protein	Verify-3D (IDScore > 0.2)	ERRAT Score	ProSA (Z Score)
Cry1AcF	92.56%	77.193	−9.69
*Helicoverpa armigera* (*Ha*APN1)	79.86%	65.8	−10.21
*Spodoptera litura* (*Sl*APN1)	83.40%	67	−10.89

**Table 3:** Analysis of protein-protein interaction (hydrogen bond interaction) in the *Ha*APN1-Cry1AcF interaction.

Donor	Chain	Amino Acid Number	Molecules Involved in H Bonding	Acceptor	Chain	Amino Acid Number	Molecules Involved in H Bonding	Distance
SER	A *	443	OG	ASN	B	494	OD1	3.19
SER	A	443	OG	ALA	B	492	O	1.86
ASN	B ^#^	494	ND2	SER	A	443	O	2.80
LYS	B	463	NZ	ASN	A	442	OD1	2.60
ASN	B	494	N	ASN	A	442	O	2.89
ASN	B	494	ND2	ASN	A	376	O	3.22
GLN	A	347	N	GLU	B	399	OE1	2.05
HSD	B	454	NDI	ALA	A	344	O	3.29
ASN	B	459	ND2	THR	A	340	OG1	3.16
THR	A	340	OG1	ASN	B	459	OD1	2.47
HSD	B	454	NE2	TYR	A	314	OH	2.96

* Cry1AcF protein—A Chain; ^#^ *HaAPN1* protein—B chain.

**Table 4:** Analysis of protein-protein interaction (H-bond interaction) in the *Sl*APN1-Cry1AcF interaction.

Donor	Chain	Amino Acid Number	Molecules Involved in H Bonding	Acceptor	Chain	Amino Acid Number	Molecules Involved in H Bonding	Distance
SER	B ^#^	220	OG	TYR	A	306	OH	2.98
SER	B	220	OG	GLN	A	320	OE1	2.54
TYR	B	213	OH	GLN	A	347	O	2.99
ARG	A *	349	NH1	THR	B	208	O	2.99
ARG	A	349	NH2	SER	B	248	O	2.21
ARG	B	269	NH1	VAL	A	351	O	3.05
ARG	B	269	NE	PRO	A	397	O	2.92
ARG	B	269	NH2	PR0	A	397	O	2.37

* Cry1AcF protein—A Chain; ^#^ *Sl*APN1 protein—B chain.

## Supplementary Materials

The following are available online at https://www.mdpi.com/2072-6651/11/3/143/s1, File S1: Secondary structure of *Ha*APN1 and *Sl*APN1 as generated by PSIPRED.
