# Supplementary material for: Molecular Interaction-Based Exploration of the Broad Spectrum Efficacy of a Bacillus thuringiensis Insecticidal Chimeric Protein, Cry1AcF
Source: Toxins (Basel). 2019 Mar 2;11(3):143. doi: 10.3390/toxins11030143 (PMC6468889; doi:10.3390/toxins11030143)
Supplement: Supplementary file 1 [file toxins-11-00143-s001.pdf]

Secondary structure of *Ha*APN1 as generated by PSIPRED

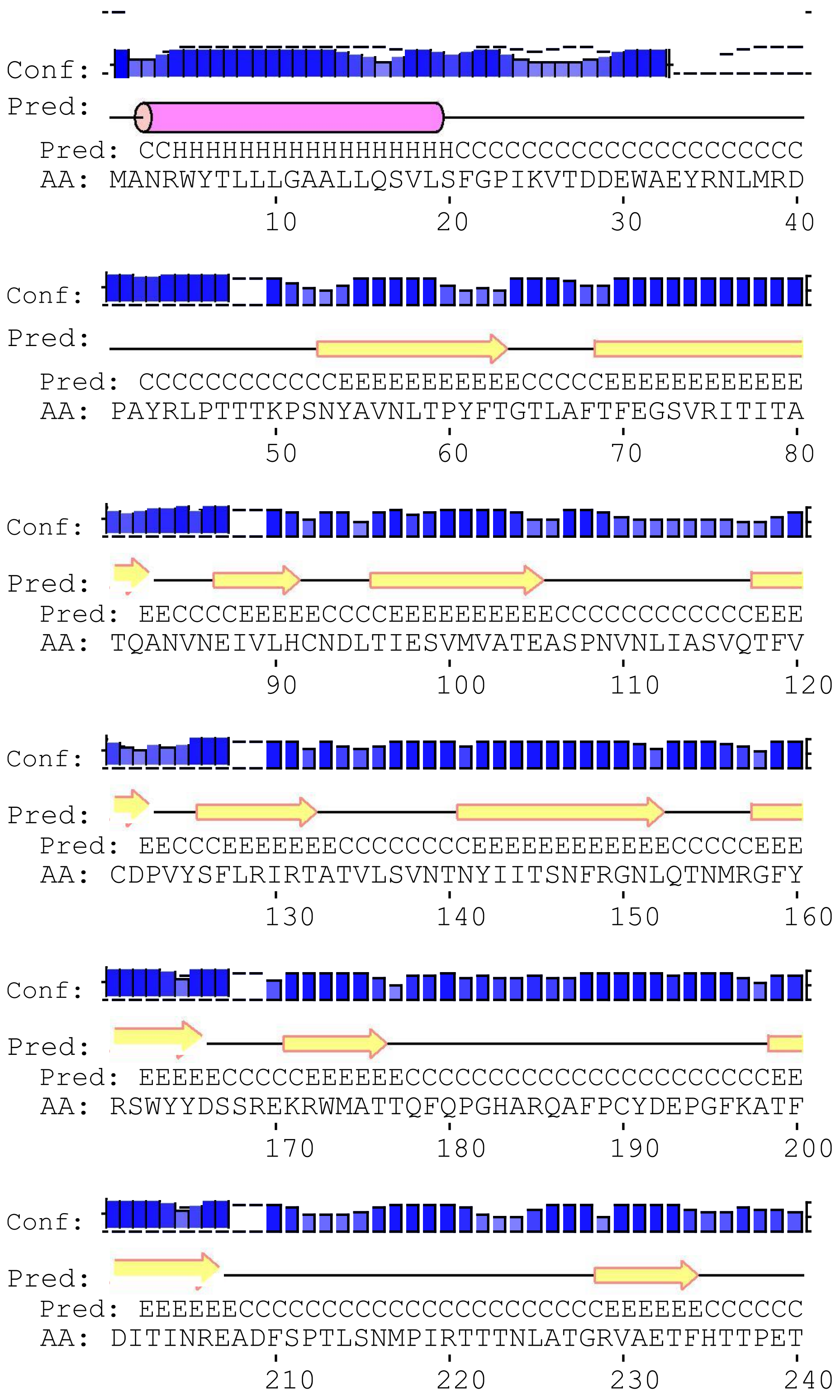

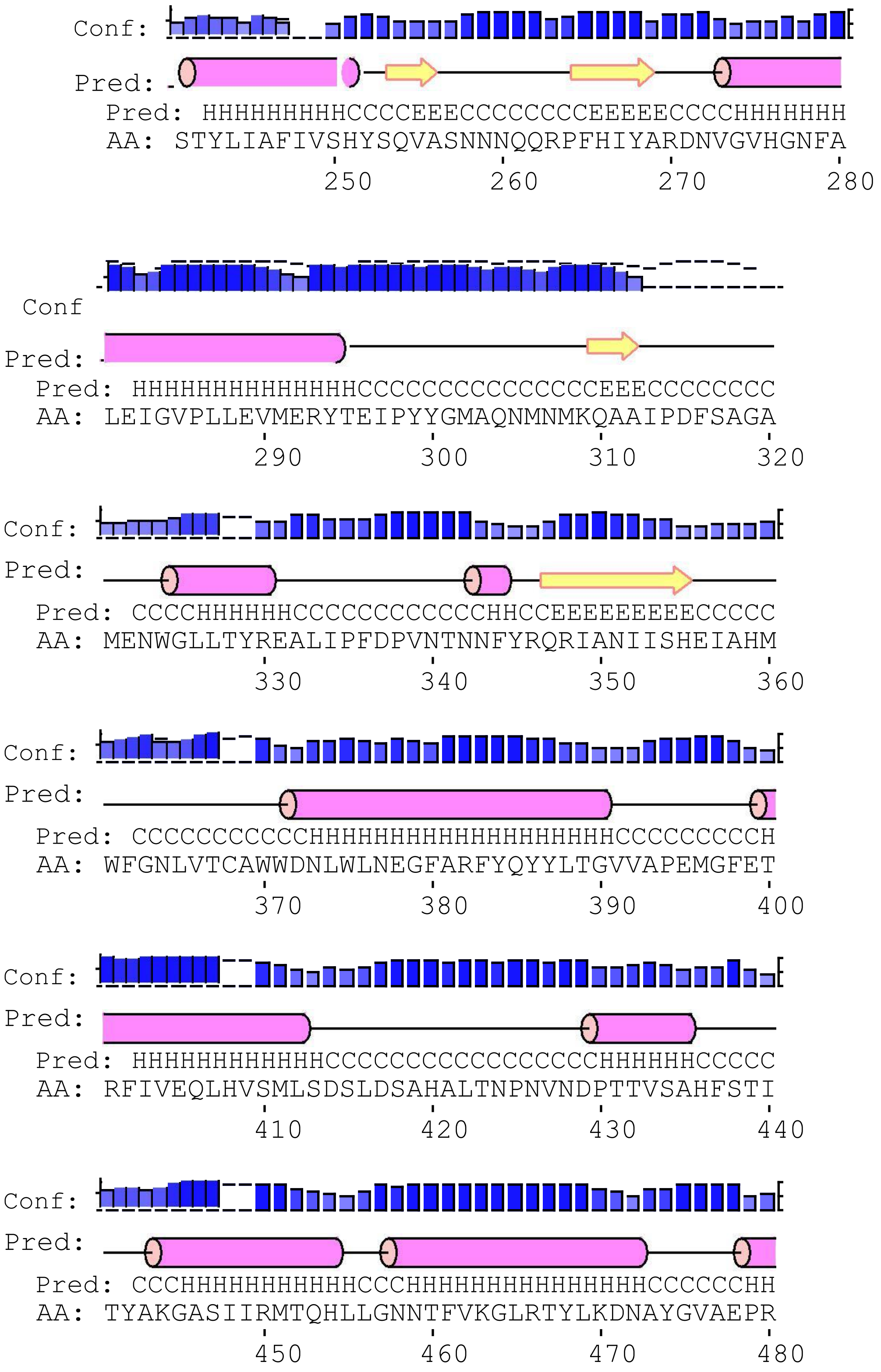
Conf:
Pred:
Pred: HHHHHHHHHCCCCEEECCCCCCCEEEEECCCCHHHHHHHH
AA: STYLIAFIVSHYSQVASNNNQQRPFHIYARDNVGVHGNFA
250 260 270 280
Conf
Pred:
Pred: HHHHHHHHHHHHHHCCCCCCCCCCCCCCCEEECCCCCCCC
AA: LEIGVPLLEVMERYTEIPYYGMAQNMNMKQAAIPDFSAGA
290 300 310 320
Conf:
Pred:
Pred: CCCCHHHHHCCCCCCCCCCCCHHCCEEEEEEEEEECCCCC
AA: MENWGLLTYREALIPFDPVNTNNFYRQRIANIISHEIAHM
330 340 350 360
Conf:
Pred:
Pred: CCCCCCCCCCCHHHHHHHHHHHHHHHHHHHCCCCCCCCCH
AA: WFGNLVTCAWWDNLWLNEGFARFYQYYLTGVVAPEMGFET
370 380 390 400
Conf:
Pred:
Pred: HHHHHHHHHHHHCCCCCCCCCCCCCCCCCHHHHHHCCCCC
AA: RFIVEQLHVSMLSDSLDSAHALTNPNVNDPTTVSAHFSTI
410 420 430 440
Conf:
Pred:
Pred: CCCHHHHHHHHHHHCCCHHHHHHHHHHHHHHHCCCCCCHH
AA: TYAKGASIIRMTQHLLGNNTFVKGLRTYLKDNAYGVAEPR
450 460 470 480

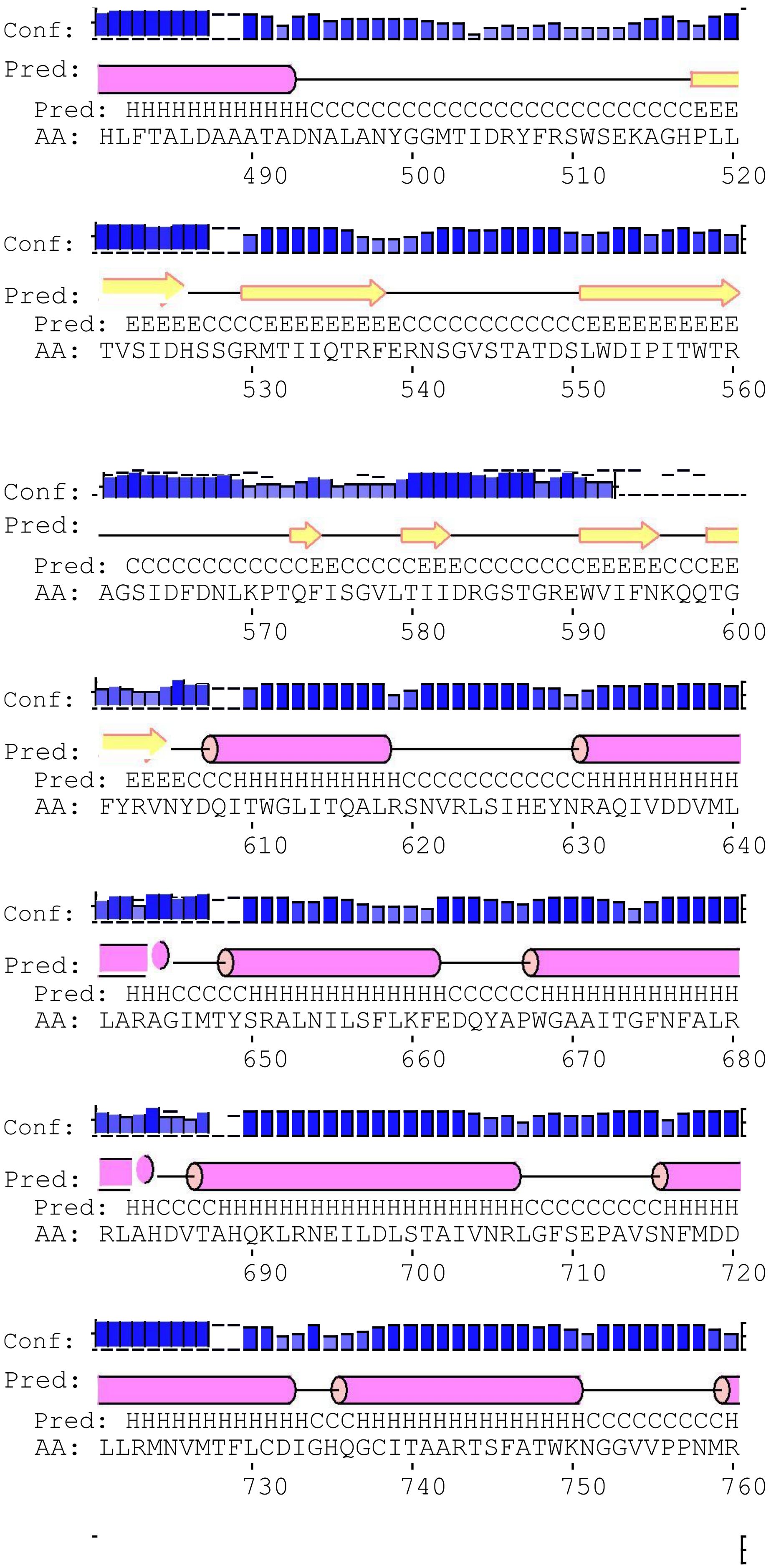
Conf:
Pred:
Pred: HHHHHHHHHHHHCCCCCCCCCCCCCCCCCCCCCCCCCEEE
AA: HLFTALDAAATADNALANYGGMTIDRYFRSWSEKAGHPLL
490 500 510 520
Conf:
Pred:
Pred: EEEEECCCCEEEEEEEEECCCCCCCCCCCCEEEEEEEEEE
AA: TVSIDHSSGRMTIIQTRFERNSGVSTATDSLWDIPITWTR
530 540 550 560
Conf:
Pred:
Pred: CCCCCCCCCCCCEECCCCCEEECCCCCCCCEEEEECCCEE
AA: AGSIDFDNLKPTQFISGVLTIIDRGSTGREWVIFNKQQTG
570 580 590 600
Conf:
Pred:
Pred: EEEECCCHHHHHHHHHHHCCCCCCCCCCCHHHHHHHHHH
AA: FYRVNYDQITWGLITQALRSNVRLSIHEYNRAQIVDDVML
610 620 630 640
Conf:
Pred:
Pred: HHHCCCCCHHHHHHHHHHHHHCCCCCCHHHHHHHHHHHHH
AA: LARAGIMTYSRALNILSFLKFEDQYAPWGAAITGFNFALR
650 660 670 680
Conf:
Pred:
Pred: HHCCCCHHHHHHHHHHHHHHHHHHHHHCCCCCCCCCHHHHH
AA: RLAHDVTAHQKLRNEILDLSTAIVNRLGFSEPAVSNFMDD
690 700 710 720
Conf:
Pred:
Pred: HHHHHHHHHHHHCCCHHHHHHHHHHHHHHHCCCCCCCCCH
AA: LLRMNVMTFLCDIGHQGCITAARTSFATWKNGGVVPPNMR
730 740 750 760

```
Conf:
Pred:
  Pred: HHHHHHHHHCCCHHHHHHHHHHHHCCCCHHHHHHHHHHHC
  AA: PWVYCNGVRYGDQSDFTHLWTSYKQSDVANDKLVMLSAAG
                      770       780       790       800

Conf:
Pred:
  Pred: CCCCHHHHHHHHHHHHCCCCCCCCCCCHHHHHHHHHCCCCC
  AA: CTLNQASLNIFLNDIVSGGDDIRPQDHSAAIVAAVRSNEV
                      810       820       830       840

Conf:
Pred:
  Pred: CHHHHHHHHHHHHHHHHHHHHCCCHHHHHHHHHHCCCHHHH
  AA: NTMRVFTWLQANVQQTISTLGSVSPILNEITARLLNEAQI
                      850       860       870       880

Conf:
Pred:
  Pred: HHHHHHHHHHHHCCCHHHHHHHHHHHHHHHHHHHHHHHHCH
  AA: TQVQTWLDANQNVIGTAAHTSATNGIATSRSNIQWYTQRV
                      890       900       910       920

Conf:
Pred:
  Pred: HHHHHHHHHCCCCCCCCCCCCCCCCCCCCCCCCCCCCCCC
  AA: PEFNVYFETGYVEENFADTTTTSTTTTTTTTTTTTAAPTT
                      930       940       950       960

Conf:
Pred:
  Pred: CCCCCCCCEEEEEECCCCCCCCCCCCCCCCCCCCHHHHHHH
  AA: TTTTEAPTTTTTTTTTEAPTTTTTEATTTPVPGSANIATLS
                      970       980       990       1000

Conf:
Pred:
Pred: HHHHHHHHHHHCC
  AA: IVTMIVTLVVNMA
                  1010
```

Legend:

= helix Conf: = confidence of prediction

\- +

= strand Pred: predicted secondary structure

= coil AA: target sequence

Secondary structure of *Sl*APN1 as generated by PSIPRED

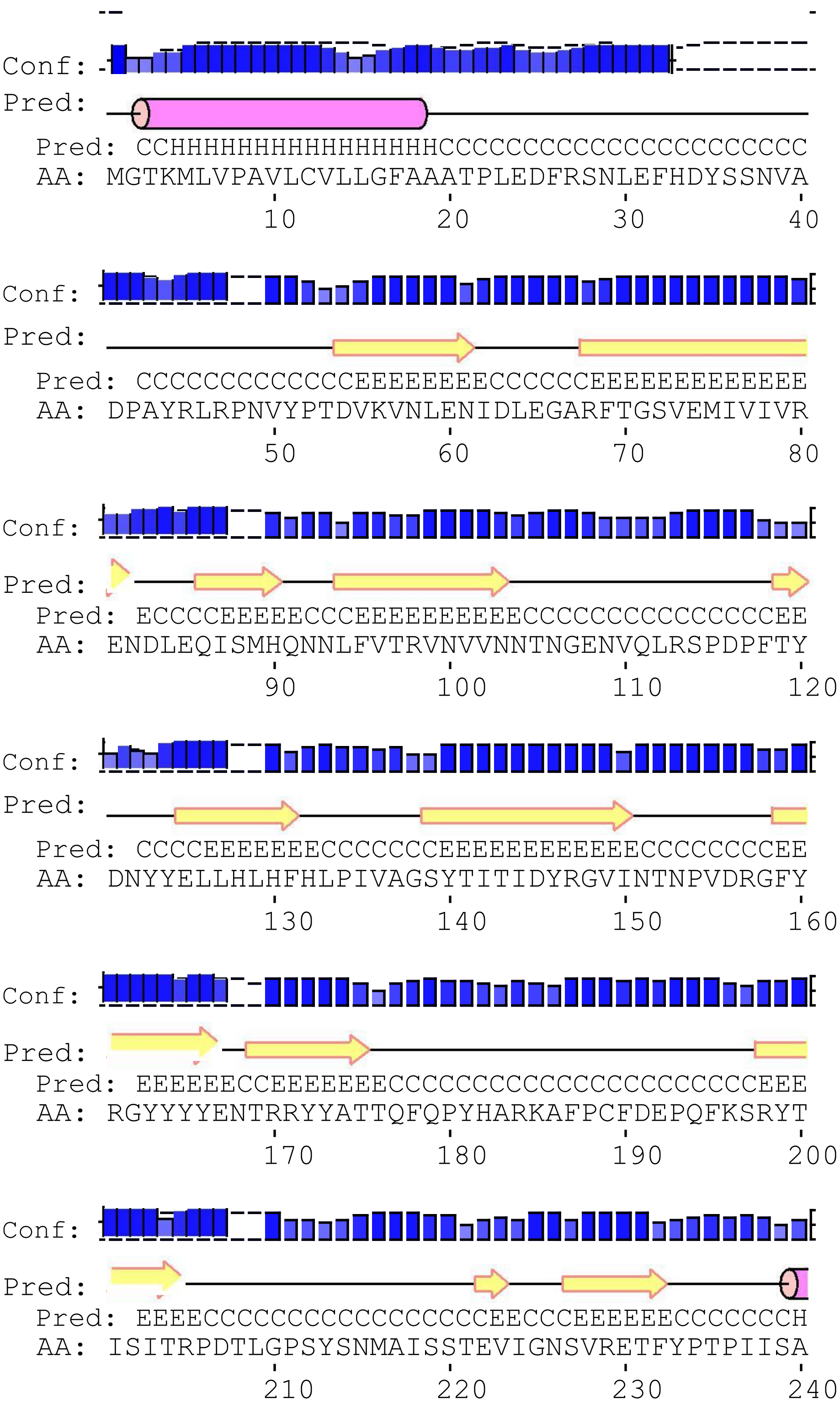

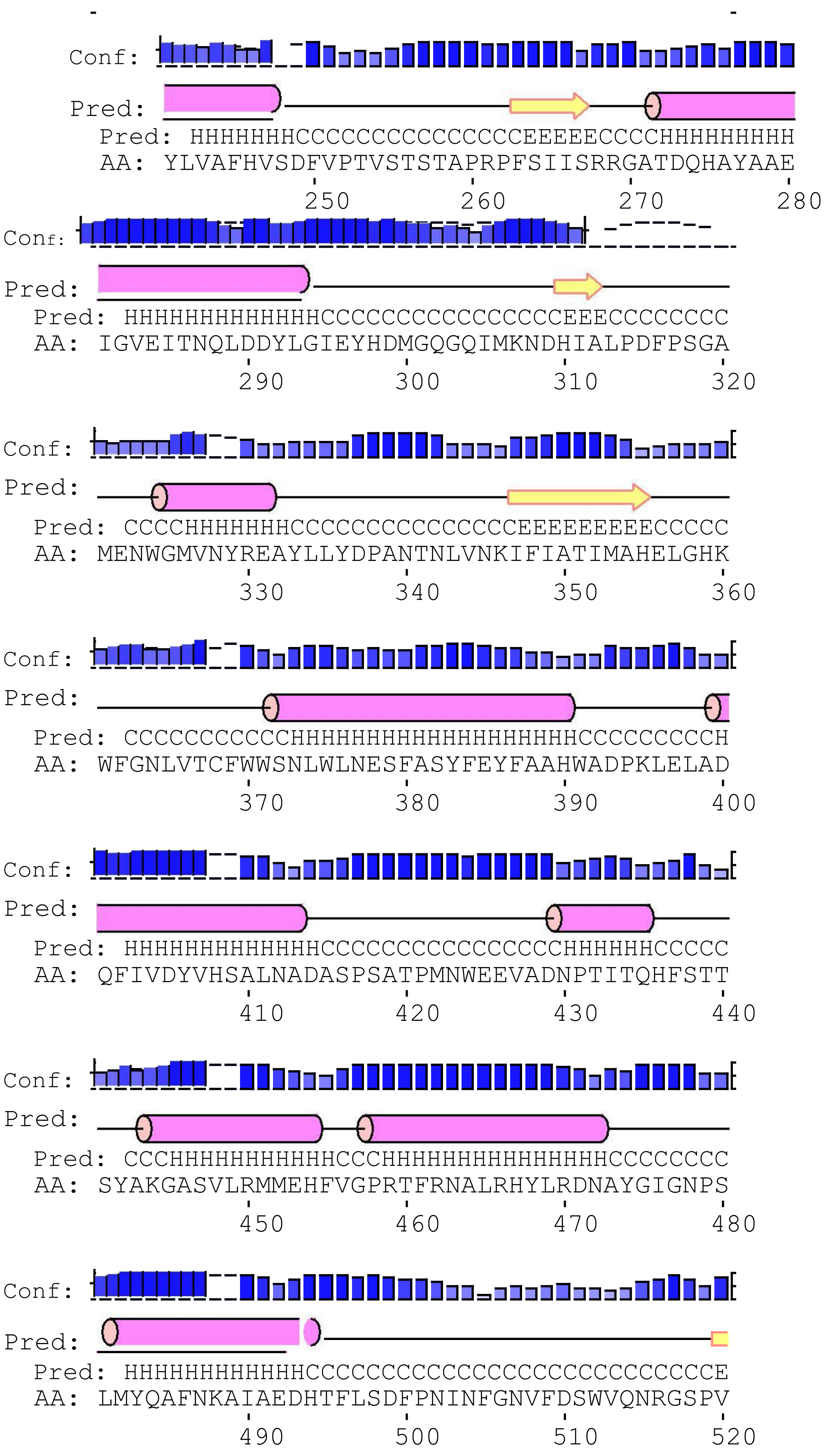

Conf:
Pred:
Pred: HHHHHHHCCCCCCCCCCCCCCCEEEEECCCCHHHHHHHHH
AA: YLVAFHVSDFVPTVSTSTAPRPFSIISRRGATDQHAYAAE
250 260 270 280
Conf:
Pred:
Pred: HHHHHHHHHHHHHCCCCCCCCCCCCCCCCEEECCCCCCCC
AA: IGVEITNQLDDYLGIEYHDMGQGQIMKNDHIALPDFPSGA
290 300 310 320
Conf:
Pred:
Pred: CCCCHHHHHHHCCCCCCCCCCCCCCCEEEEEEEEECCCCC
AA: MENWGMVNYREAYLLYDPANTNLVNKIFIATIMAHELGHK
330 340 350 360
Conf:
Pred:
Pred: CCCCCCCCCCCHHHHHHHHHHHHHHHHHHHCCCCCCCCCH
AA: WFGNLVTCFWWSNLWLNESFASYFEYFAAHWADPKLELAD
370 380 390 400
Conf:
Pred:
Pred: HHHHHHHHHHHHHCCCCCCCCCCCCCCCCHHHHHHCCCCC
AA: QFIVDYVHSALNADASPSATPMNWEEVADNPTITQHFSTT
410 420 430 440
Conf:
Pred:
Pred: CCCHHHHHHHHHHHCCCHHHHHHHHHHHHHHHCCCCCCCC
AA: SYAKGASVLRMMEHFVGPRTFRNALRHYLRDNAYGIGNPS
450 460 470 480
Conf:
Pred:
Pred: HHHHHHHHHHHHCCCCCCCCCCCCCCCCCCCCCCCCCCCE
AA: LMYQAFNKAIAEDHTFLSDFPNINFGNVFDSWVQNRGSPV
490 500 510 520

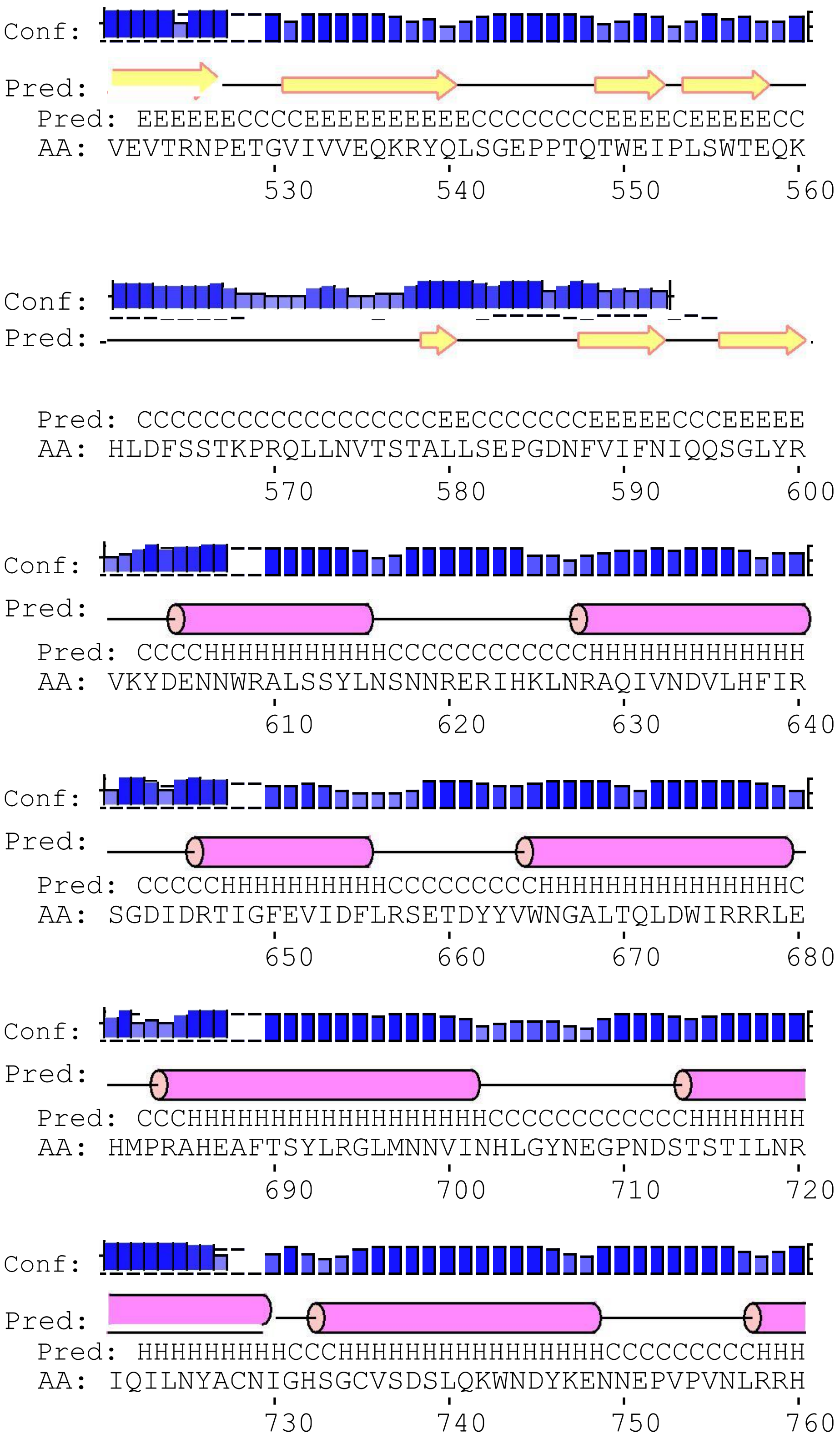
Conf:
Pred:
Pred: EEEEEECCCCEEEEEEEEEECCCCCCCCEEEECEEEEECC
AA: VEVTRNPETGVIVVEQKRYQLSGEPPTQTWEIPLSWTEQK
530 540 550 560
Conf:
Pred:
Pred: CCCCCCCCCCCCCCCCCCEECCCCCCCEEEEECCCEEEEE
AA: HLDFSSTKPRQLLNVTSTALLSEPGDNFVIFNIQQSGLYR
570 580 590 600
Conf:
Pred:
Pred: CCCCHHHHHHHHHHHCCCCCCCCCCCCHHHHHHHHHHHHH
AA: VKYDENNWRALSSYLNSNNRERIHKLNRAQIVNDVLHFIR
610 620 630 640
Conf:
Pred:
Pred: CCCCCHHHHHHHHHHCCCCCCCCCHHHHHHHHHHHHHHHC
AA: SGDIDRTIGFEVIDFLRSETDYYVWNGALTQLDWIRRRLE
650 660 670 680
Conf:
Pred:
Pred: CCCHHHHHHHHHHHHHHHHHHCCCCCCCCCCCCHHHHHHH
AA: HMPRAHEAFTSYLRGLMNNVINHLGYNEGPNDSTSTILNR
690 700 710 720
Conf:
Pred:
Pred: HHHHHHHHHCCCHHHHHHHHHHHHHHHHCCCCCCCCCHHH
AA: IQILNYACNIGHSGCVSDSLQKWNDYKENNEPVPVNLRRH
730 740 750 760

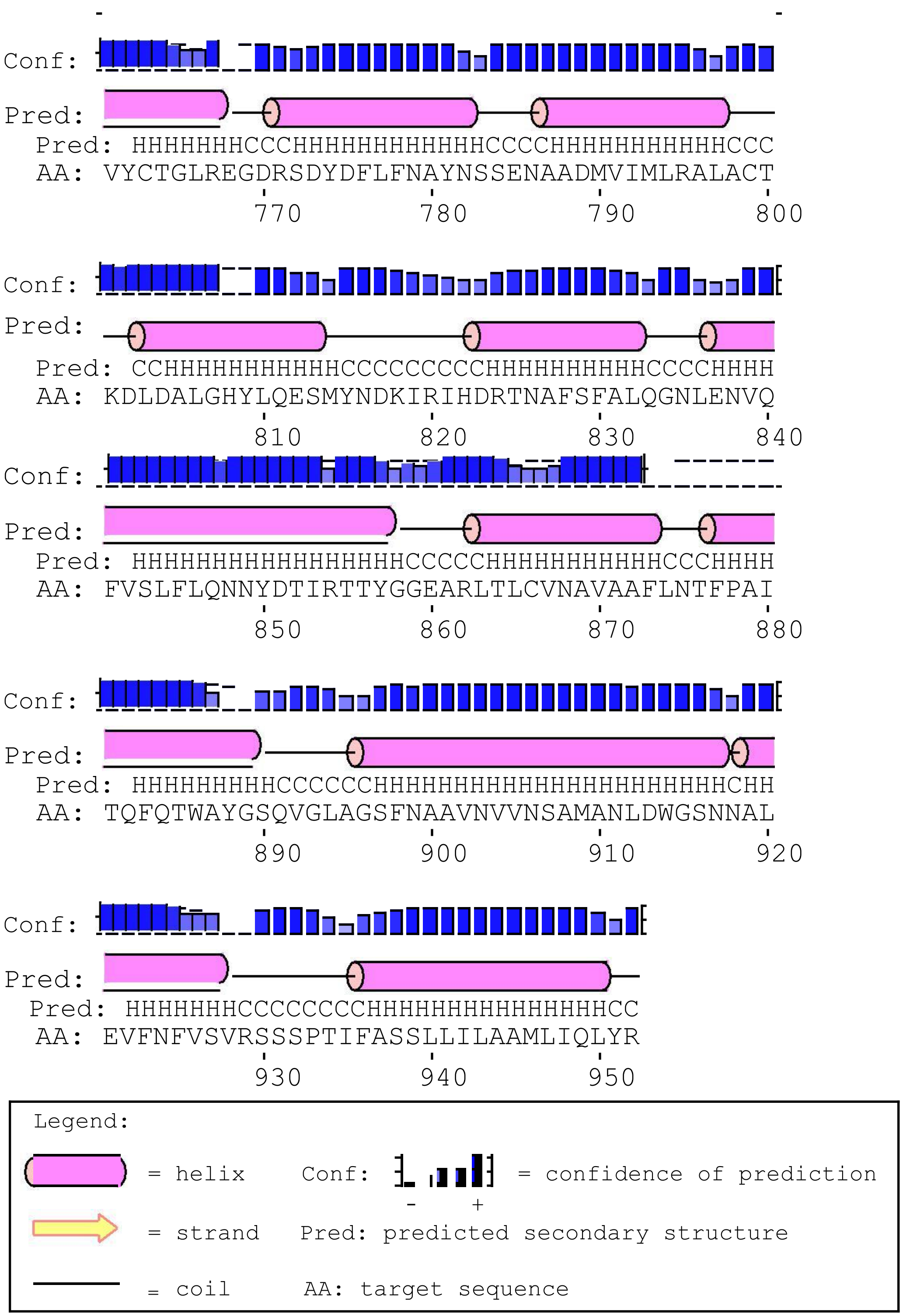

Conf:
Pred:
Pred: HHHHHHHCCCHHHHHHHHHHHHHCCCCHHHHHHHHHHHHHCCC
AA: VYCTGLREGDRSDYDFLFNAYNSSENAADMVIMLRALACT
770 780 790 800
Conf:
Pred:
Pred: CCHHHHHHHHHHHHCCCCCCCCCHHHHHHHHHHHCCCCHHHH
AA: KDLDALGHYLQESMYNDKIRIHDRTNAFSFALQGNLENVQ
810 820 830 840
Conf:
Pred:
Pred: HHHHHHHHHHHHHHHHHHCCCCCHHHHHHHHHHHHCCCHHHH
AA: FVSLFLQNNYDTIRTTYGGEARLTLCVNAVAAFLNTFPAI
850 860 870 880
Conf:
Pred:
Pred: HHHHHHHHHCCCCCCHHHHHHHHHHHHHHHHHHHHHHHCHH
AA: TQFQTWAYGSQVGLAGSFNAAVNVVNSAMANLDWGSNNAL
890 900 910 920
Conf:
Pred:
Pred: HHHHHHHCCCCCCCCHHHHHHHHHHHHHHHHCC
AA: EVFNFVSVRSSSPTIFASSLLILAAMLIQLYR
930 940 950
Legend:
= helix
Conf:
= confidence of prediction
- +
= strand
Pred: predicted secondary structure
= coil
AA: target sequence
